# Do not harm: a minimal defense of conscientious objection in the medical profession

**DOI:** 10.1007/s11017-026-09759-0

**Published:** 2026-05-20

**Authors:** Darrin W. Snyder Belousek

**Affiliations:** Department of English, Philosophy, and Religion, Ohio Northern University, 525 S. Main St.,, Ada, OH 45810 USA

**Keywords:** Conscientious objection, Medical profession, Conscience-based refusal, Incompatibility thesis

## Abstract

I defend conscientious objection (CO) in the medical profession by refuting the incompatibility thesis (IT). IT maintains that CO is incompatible with fulfilling the objectives and obligations of the medical profession and thus impermissible for medical professionals. To refute IT, I construct a case of conscience-based refusal in parallel with a case of profession-based refusal, such that both cases fulfill the primary objective and core obligation of the medical profession, thereby directly demonstrating the compatibility of CO with fulfilling obligations in the medical profession. I conclude that IT is false and thus that CO is permissible for medical professionals. I then defend my argument against potential objections. My argument strategy is two-fold. First, to avoid begging the question, I follow a neutral definition of CO that is compatible with IT. Second, to make the argument as logically strong as possible, I assume only minimal claims about the primary objective and core obligation of the medical profession that should be acceptable to (at least some) defenders of IT.

## Introduction: conscientious objection and the incompatibility thesis in the medical profession

The ongoing debate over conscientious objection (CO) in the health care professions has generated three general positions: conscience absolutism, incompatibility, and compromise [1]. The first two positions represent the extremes of the debate. Conscience absolutism maintains that “health care professionals generally do not have an obligation to perform *any action*, including disclosure and referral, contrary to their conscience,” such that conscientious objection should be permissible for health care professionals, under any conditions and without any qualifications [1, p. 33]. Conscience incompatibilism maintains that “conscience-based refusals to provide legal and professionally permitted goods and services within the scope of a practitioner’s competence are incompatible with the practitioner’s professional obligations,” such that conscientious objection should be impermissible for health care professionals, under (almost) any conditions and without (almost) any qualifications [1, p. 34]. Conscience compromise, which seems to be the majority position in the debate, maintains that health care professionals may exercise conscience-based refusals, under certain conditions (e.g., not causing harm or imposing undue burden on patients) and with certain obligations (e.g., disclosing options and referring patients).

I focus in this paper on the conscience incompatibilism position. Following Wicclair [1], I will call the assumption that a conscience-based refusal to provide legal and professionally permitted goods and services within the scope of the practitioner’s competence is incompatible with the practitioner’s professional obligations the ‘incompatibility thesis’ (IT). The IT can be used to construct an argument against the permissibility of CO in the medical profession, which I will call the impermissibility syllogism:

- *Premise 1*: Any form of medical practice that is incompatible with fulfilling the medical practitioner's professional obligations is impermissible in the medical profession.
- *Premise 2*: CO in medical practice is incompatible with fulfilling the medical practitioner's professional obligations. (IT)
- *Conclusion*: Therefore, CO in medical practice is impermissible in the medical profession.

The IT is not merely a theoretical proposition about professional obligation but has practical implications for the career prospects of health care professionals. The IT can also be used to construct what I will call the unacceptability syllogism:

- *Premise 1*: A medical practitioner must either fulfill their professional obligations or leave the medical profession.^1^
- *Premise 2*: A medical practitioner exercising CO fails to fulfill their professional obligations. (IT)
- *Conclusion*: Therefore, a medical practitioner exercising CO must leave the medical profession.

Conscience incompatibilism maintains not only that conscientious objection is fundamentally incompatible with fulfilling professional obligations, but also that conscientious objectors are generally unacceptable within the medical profession. Conscience incompatibilism thus rejects (nearly) any toleration of conscience-based claims in medical practice and (nearly) any accommodation of medical professionals wishing to exercise conscience-based refusal.

Although not the majority position, conscience incompatibilism is not merely the view of a marginal few. The IT, along with the impermissibility and unacceptability syllogisms premised on it, has been endorsed by mainstream figures in medicine and bioethics holding prominent and influential positions and published in major medical and bioethical journals. For example, as Julian Savulescu has argued:

A doctors’ conscience has little place in the delivery of modern medical care. What should be provided to patients is defined by the law and consideration of the just distribution of finite medical resources, which requires a reasonable conception of the patient’s good and the patient’s informed desires. If people are not prepared to offer legally permitted, efficient, and beneficial care to a patient because it conflicts with their values, they should not be doctors. Doctors should not offer partial medical services or partially discharge their obligations to care for their patients [4, p. 294]

Although Savulescu argues that CO is incompatible with the medical profession, he allows for toleration of CO when it “can be accommodated without compromising the quality and efficiency of public medicine,” but maintains that CO should not be tolerated if it “compromises the quality, efficiency, or equitable delivery of a service” [4, p. 296].^2^

As Ronit Stahl and Ezekiel Emanuel have argued:

To invoke conscientious objection is to reject the fundamental obligation of health care—the primary duty to ensure patients’ continued well-being. It places a professional’s personal beliefs above professional standards….The health care professional who wants to prioritize personal values over professional duties must choose a less personally fraught occupation [5, pp. 1383-1384].

While Stahl and Emanuel argue that CO is incompatible with professional obligation, they maintain a narrow allowance for CO restricted to “professionally contested interventions” (including physician-assisted suicide, but excluding abortion), with obligations of disclosure and referral [5, p. 1383]. In their view, health care professionals unwilling to accept these restrictions on conscience must either relocate to a medical field where they will not encounter conflicts with their conscience or remove themselves from the medical profession entirely.

And as Udo Schuklenk and Ricardo Smalling have argued:

Medical professionals have no moral claim in liberal democratic societies to the accommodation of their individual conscientious objections. To accommodate such objections would subvert some of the very reasons for why the medical profession was created in the first place. To accommodate them would also permit such medical professionals to abuse the monopoly privileges that society endowed their profession with. Medical professionals practicing medicine in the 21st century would be well advised to accept professional service delivery, as defined by the scope of professional practice, as one inevitable corollary of their voluntary decision to join the profession [6, p. 240].

Schuklenk and Smalling, while generally rejecting toleration of conscience claims and accommodation of conscientious objectors in the medical profession, suggest allowing accommodation of long-term practitioners who entered the profession under “a different kind of contract” but state that such practitioners have no moral right to accommodation within the profession [6, p. 239].

## Preliminaries: aims, means, definitions, and principles

### Aims and means

My purpose in this paper is to make a ‘minimal’ defense of CO in the medical profession, a defense that is minimal in both aim and means. First, I aim to defend CO in the medical profession to the logically minimal extent possible simply by refuting IT: demonstrating that IT is false also demonstrates that the impermissibility and unacceptability syllogisms are unsound, which thus defends the claim that CO is permissible. Second, I seek to achieve this minimal aim by minimal means. I seek to refute IT and defend CO by means independent of religious beliefs, metaphysical claims, or political theories. To do this I will construct a case where CO is evidently compatible with fulfilling professional obligations, which will at once demonstrate the falsity of IT and defend the permissibility of CO.

My approach to refuting IT and demonstrating the compatibility of CO with fulfilling professional obligations in the medical profession differs from previous approaches. Mark Wicclair has examined several arguments presented in support of the incompatibilism position, arguing that all these arguments fall short of conclusively establishing IT. The inconclusiveness of the incompatibilism arguments leaves room for a compromise position, which Wicclair favors and develops [1]. Similarly, Xavier Symons has examined the theoretical assumptions made by several conscience incompatibilists about the nature of the medical profession, the role of the medical practitioner, and the physician–patient relationship, arguing that these various assumptions are problematic or unsupported [2].

Rather than critiquing arguments for incompatibilism and the assumptions of incompatibilists, I take a direct approach to refuting incompatibilism by constructing a case to demonstrate that IT is false. In making my argument and constructing the case, to avoid begging the question, I aim to depend on only minimal assumptions that should be generally acceptable and potentially agreeable to a conscience incompatibilist. My argument will not depend on assuming a substantive theory of wellbeing or a comprehensive theory of health. My argument will not invoke metaphysical claims about the nature of conscience or moral agency and integrity, nor religious claims about the sanctity of conscience, nor political claims about individual rights and liberties. Here my approach in this paper differs again from Xavier Symons [2]. He develops a theory of conscience and argues that conscience inevitably, and appropriately, plays a vital role in the medical profession, conceptualized as a virtue- and character-based practice. If Symons’ views of the nature of conscience, the medical profession, and medical judgment are correct, then IT is a nonstarter: CO is compatible with fulfilling professional obligations because conscience is integral to good medical judgment. While I’m sympathetic to Symons’ approach, the conscience incompatibilist might disagree with its metaphysical and moral assumptions and thus consider his approach to beg the question.

### Definitions

Several key terms I employ in the argument need to be defined for the context of this argument. First, I adopt Mark Wicclair’s definitions of CO and its associated terms [1]. These definitions do not depend on any theory of the nature of conscience and are neutral between differing positions on the compatibility of CO with the medical profession. Wicclair defines conscientious objection as follows: A medical professional engages in an act of conscientious objection when they:

1. Refuse to provide legal and professionally accepted goods or services within the scope of their professional competence; and
2. Justify their refusal by claiming that it is an act of conscience or is conscience-based.

Wicclair defines conscience-based refusal as follows: An agent’s refusal to provide a good or service is conscience-based if and only if:

1. The agent has a core set of moral (i.e., ethical or religious) beliefs;
2. Providing the good or service is incompatible with the agent’s core moral beliefs; and
3. The agent’s refusal is based on their core moral beliefs.

Wicclair defines core moral beliefs as “an agent’s fundamental moral beliefs,” comprising “the subset of an agent’s moral beliefs that matter most to the agent” [1, p. 4] Core moral beliefs are integral to an agent’s self-understanding (i.e., self-conception or identity), such that “acting contrary to core moral beliefs is perceived by the agent as an act of self-betrayal” [1, p. 5]. Core moral beliefs may, but need not, be based on religious beliefs.

Second, I adopt also Wicclair’s distinction between conscience-based refusal and profession-based refusal. Wicclair defines profession-based refusals as “Refusals based on considerations of professional integrity,” which “are grounded in professional norms and standards—or a provider’s understanding of them” [1, p. 6]. Profession-based refusals are distinguishable from conscience-based refusals: “Insofar as refusals are based on a provider’s understanding of professional norms and standards rather than her personal ethical or religious beliefs, they are distinguishable from conscience-based refusals” [1, p. 6]. Such profession-based refusals serve to uphold the norms and standards of the medical profession and to safeguard the practitioner’s integrity as a medical professional. The key difference between a conscience-based refusal and a profession-based refusal is the grounding of the considerations on which the refusal is based: in a conscience-based refusal, the agent grounds the reason on which their refusal is based in their personal core moral beliefs; in a profession-based refusal, the agent grounds the reason on which their refusal is based in professional norms and standards.

Third, the notion of health harm and benefit also figures in the argument and needs to be defined here. In my argument, health benefit or harm will refer to a benefit or harm to a patient’s physical health. Health benefit refers to alleviating or removing a pained, injured, ill, diseased, or dysfunctional condition in a patient, relative to the patient’s prior condition. Health harm refers to something that causes or worsens a pained, injured, ill, diseased, or dysfunctional condition in a patient, relative to the patient’s prior condition.

A couple caveats: I take these definitions of health benefit and harm to be adequate for the purposes of this argument, not necessarily to be adequate as final definitions. These definitions assume that health is a specific aspect of overall human wellbeing but do not assume a comprehensive theory of human wellbeing or health. The notion of health benefit and harm I am using here is an objective notion: health benefit or harm is not mere satisfaction or frustration of patient desires or preferences. Thus, a physician’s treatment of a patient’s condition could benefit a patient’s health by alleviating or removing an injury or disease, even if the treatment did not accord with the patient’s wishes.^3^ Likewise, a physician’s refusal of a patient’s request by itself does not harm the patient’s health apart from empirically demonstrable and expertly diagnosable consequences (pain, injury, illness, disease, or dysfunction) for the patient’s physical condition, even if the physician’s refusal frustrates the patient’s desires or fails to satisfy the patient’s preferences.

### Principles

The argument will rely on several principles, related to personal morality and medical ethics, that need to be explicated.

### Principles of personal morality

First, two principles related to personal morality: a core belief and a fundamental duty. The following is a core moral belief, common to most reasonable people: it is morally wrong to knowingly and willfully harm another person. This core moral belief grounds a fundamental personal moral duty: Do not harm other persons.

For this argument, I do not assume any deeper grounding of this core belief and fundamental duty in religious claims, universal principles, or general theories of morality. Most reasonable people who hold this belief and act on this duty, I assume, do so without thinking they need to appeal to deeper reasons to justify doing so.

Most reasonable people, I assume also, would think that the fundamental duty is not an absolute duty. They would think that it is sometimes morally permissible to violate the duty not to harm other persons, such as when an action to help someone in need would foreseeably result in harm to them or others. For this argument, therefore, I take this fundamental moral duty as a prima facie duty: one always has strong moral reason to act on it; but it can be overridden by stronger moral considerations in each circumstance.

I assume further, however, that most reasonable people would think that such overriding moral considerations must meet certain criteria for violating the fundamental duty to be permissible. The following criteria seem reasonable and, I think, would be agreeable to most people: An action to help another person in need that foreseeably would result in harm to others is morally permissible only if: the agent aims to help but not to harm; and some resultant harm to others is practically unavoidable in providing the needed help by available means.^4^ These criteria can be grounded in the core belief and fundamental duty: if generally it is morally wrong to knowingly and willingly harm other persons, such that one ought never harm other persons, then one ought not aim to harm others and ought to avoid harming others if one can when providing needed help.

### Principles of medical ethics

Second, two principles connected to the medical profession: its primary objective and a core obligation. The medical profession has a primary objective, a moral good at which medical practice properly aims: to restore and maintain patient health. Accordingly, medical practice has a core obligation: do not harm patient health. Here I take this core obligation as a prima facie duty: a physician always has strong reason to act on it; but it can be overridden by stronger moral considerations in each circumstance.^5^

This core obligation of medical practice could be grounded in either of two ways. It could be grounded in a moral principle external to medical practice. The core professional obligation ‘do not harm patient health’ could be grounded in the core belief and fundamental principle of personal morality ‘do not harm other persons’ as applied to medical practice: if generally one ought never knowingly and willingly harm other persons, then a medical practitioner ought never knowingly and willingly harm a patient’s health.^6^ This core obligation could also be grounded in the internal morality of medical practice. The professional obligation “do not harm patient health” could be grounded in the primary objective of the medical profession: if medical practitioners ought always to aim at restoring and maintaining their patients’ health, then they ought never knowingly and willingly harm their patients’ health.

As a prima facie duty, it is sometimes permissible to violate this core professional obligation of medical practice in certain circumstances, such as when implementing a medical intervention to alleviate a patient’s diseased or injured condition involves causing or risking harm to the patient’s health. Permissible violation of this core obligation of the medical profession, however, is constrained by the primary objective of the medical profession: moral considerations sufficient to override the medical practitioner’s duty to not harm patient health must substantively cohere with the moral aim of benefiting patient health. Thus, alleviating a patient’s diseased or injured condition by implementing a medical intervention that foreseeably would cause or risk harm to the patient’s health is permissible only when certain criteria are met. The following criteria seem reasonable and, I think, would be agreeable to most medical practitioners: the medical practitioner aims to benefit but not to harm patient health; and some resultant harm to patient health is practically unavoidable in providing the benefit needed for patient health by available medical interventions.

As was the core obligation of medical practice, these necessary conditions of the permissibility of implementing a harm-causing/risking medical intervention could be grounded either externally in the core belief and fundamental duty of personal morality, or internally in the primary objective and core obligation of the medical profession. These specific criteria of permissibility could be grounded in the general criteria of moral permissibility as applied to medical practice: if generally one may help others in need by actions that would foreseeably result in harm to others only if one aims to help but not to harm, and some resultant harm is practically unavoidable in providing the needed help by available means, then a medical practitioner may alleviate a patient’s diseased or injured condition by a medical intervention that would foreseeably cause or risk harm to the patient’s health only if they aim to benefit but not to harm the patient’s health and some resultant harm to the patient’s health is practically unavoidable in benefitting the patient’s health by available medical interventions. These criteria of permissibility could also be grounded in the primary objective and core obligation of medical practice: if medical practitioners ought always to aim at restoring and maintaining their patients’ health and thus ought never knowingly and willingly harm their patients’ health, then they ought not aim to harm a patient’s health and ought avoid harming a patient’s health if they can when implementing medical interventions to alleviate the patient’s diseased or injured condition.

These criteria for implementing a harm-causing/risking medical intervention can be formulated and elaborated as necessary conditions of permissibility^7^:

*Permissibility Conditions*: Implementing a harm-causing/risking medical intervention is morally permissible only if:

- The medical practitioner aims to benefit but not to harm patient health; and.
- Some harm resulting from medical intervention to benefit patient health is practically unavoidable as judged by medical expertise, including at least the following:
- Demonstration of health need: diagnosed condition (disease, dysfunction, illness, infection, injury, pain, tumor, etc.), actual (present) or potential (predicted), based on empirical evidence; and
- Expectation of health benefit: the medical intervention is reasonably expected to be effective in alleviating the diagnosed condition; and
- Unavoidability of health harm: alleviating the diagnosed condition by available medical interventions cannot avoid causing/risking some harm to patient health.

Based on these permissibility conditions, I formulate a principle of impermissibility of medical intervention.

*Impermissibility Principle*: Implementing a harm-causing/risking medical intervention is morally impermissible if it fails to meet at least one necessary condition of permissible violation of the core obligation of medical practice: either the medical practitioner does not aim to benefit patient health or aims to harm patient health, or some resultant harm from medical intervention to benefit patient health is not practically unavoidable.

A patient’s desire or preference for a harm-causing/risking medical intervention, if not sought for the primary objective of patient health, or if not demonstrably needed for patient health as judged by medical expertise, is not a consideration sufficient to override the prima facie duty of the core obligation. A harm-causing/risking medical intervention that fails to satisfy either necessary condition is morally impermissible even if a patient wants and consents to the intervention.

Finally, based on this principle of impermissibility, I formulate a principle of objectionability to medical intervention. Impermissible violation of the core obligation on the grounds of either necessary condition is a sufficient condition for the moral permissibility of objection to a medical intervention.

*Objectionability Principle*: If implementing a harm-causing/risking medical intervention would fail to meet the necessary conditions of permissible violation of the core obligation of medical practice, either because the medical intervention would not be aimed at the primary objective of patient health or because causing/risking harm to patient health would not be practically unavoidable to benefitting patient health, then a medical practitioner may object to providing the intervention and refuse a patient’s request for the intervention, whatever the patient’s reasons.^8^

### Examples of the principles applied

I will illustrate how these principles of impermissibility and objectionability would apply in medical practice with simple examples. Each example involves the same medical intervention, appendectomy, which is a legal and professionally accepted service within the scope of professional competence of general surgeons and is also a harm-causing/risking medical intervention involving non-trivial risks to patient health.^9^ The examples are tailored to illustrate each permissibility condition in sequence.

First example: Patient A visits a physician and requests an appendectomy. Asked why he is requesting an appendectomy when he appears completely healthy, the patient tells the physician that, since studying human anatomy at university, he has been fascinated with having his appendix preserved in a jar for him to observe at home; aware that one can be healthy without an appendix, he wants his appendix removed and preserved. Seeing that the patient’s request is not for the sake of restoring or maintaining the patient’s health, the physician judges that performing the appendectomy would not be aimed at benefitting the patient’s health and thus would violate permissibility condition (1), such that performing the appendectomy is impermissible and objection is permissible. Accordingly, the physician simply refuses the patient’s request.

Second example: Patient B visits a physician and requests an appendectomy. Asked why she is requesting an appendectomy when she appears completely healthy, the patient tells the physician that she worries that her appendix might become infected; aware that an infected appendix might rupture and lead to sepsis, which could be fatal, she wants her appendix removed so she can worry less and be happier. Observing that the patient exhibits no symptoms of appendicitis, has no medical history of appendicitis, and does not have any risk factors for developing appendicitis, the physician judges that, while the patient’s request is (hypothetically) health-aimed, performing the appendectomy would violate permissibility condition (2a), because there is no empirical evidence of a health need (actual or potential), such that performing the appendectomy is impermissible and objection is permissible. Accordingly, the physician refuses the patient’s request but recommends that she consider seeing a therapist to help her worry less.

Third example: Patient C visits a physician, complaining of sudden, severe, and worsening abdominal pain. Recalling that his brother experienced similar symptoms and suffered a ruptured appendix, he believes his appendix is infected and requests an appendectomy. After examining the patient and running the indicated tests, which show no evidence of an inflamed appendix, the physician rules out acute appendicitis as the probable cause. Unconvinced by the evidence, the patient adamantly insists on receiving an appendectomy. Although the patient’s request is health-aimed and the patient exhibits an unhealthy condition, the physician judges that performing the appendectomy would violate permissibility condition (2b), because it would not be reasonably expected to alleviate the unhealthy condition, such that performing the appendectomy is impermissible and objection is permissible. Accordingly, the physician refuses the patient’s request but proposes to the patient testing for other possible causes, such as pseudoappendicitis and other ailments whose symptoms mimic appendicitis.

Fourth example: Patient D visits a physician, complaining of abdominal pain. After examining the patient and running the indicated tests, the physician diagnoses appendicitis but considers it a mild case detected early enough to be treated successfully with antibiotics—and thus writes a prescription for the patient rather than scheduling her for surgery. Unsatisfied with the physician’s diagnosis and proposed treatment, the patient requests an appendectomy. Although the patient’s request is health-aimed and the patient has a diagnosed condition, and although appendectomy would be reasonably expected to alleviate the diagnosed condition, the physician judges that performing the appendectomy would violate permissibility condition (2c), because the patient’s diagnosed condition could be alleviated by antibiotics without the health risks of surgery, such that performing the appendectomy is impermissible and objection is permissible. Accordingly, the physician refuses the patient’s request but offers to refer her to another physician for a second opinion.^10^

## Argument: conscientious objection is compatible with professional obligation

I will now argue, premised on the definitions and principles above, that CO is compatible with fulfilling professional obligations, and thus that IT is false, by directly demonstrating the compatibility of CO with fulfilling professional obligations.

### The logic of the argument

The IT is a universal statement that excludes CO in medical practice from compatibility with fulfilling professional obligations: if IT is true, then no instance of CO in medical practice is compatible with fulfilling professional obligations. As a universal statement, however, IT is logically refutable by a single counterinstance: if there is any instance of CO in medical practice that is compatible with fulfilling professional obligations, then IT is false. Here I construct a case that, I argue, is an instance of CO in medical practice that is compatible with fulfilling obligations in the medical profession, thereby directly refuting IT.

My strategy in constructing the counterinstance to IT is to construct parallel cases using the definitions and principles set forth above. I first construct a case of profession-based refusal that is evidently compatible with the primary objective and professional obligations of medicine. I then construct a parallel case of conscience-based refusal that is arguably analogous to the case of profession-based refusal.

### A case of profession-based refusal

Consider Physician A. As a medical professional, she is committed to the primary objective of the medical profession; her aim in practicing medicine is to restore and maintain patient health. She grounds the core obligation of medical practice—‘Do not harm patient health’—in the primary objective of the medical profession.

Physician A receives a patient’s request for a legal and professionally accepted service within the scope of her professional competence, such as one of the patient requests in the examples above. Considering the impermissibility principle, she judges that providing the requested service would fail to satisfy the necessary conditions for the permissible violation of the core obligation of medical practice—and, thus, judges that providing the requested service would be incompatible with the primary objective of the medical profession. Invoking the objectionability principle, she concludes that objection to the patient’s request is morally permissible and, grounding her objection in the core professional obligation, refuses to provide the requested service. She claims that her refusal upholds the primary objective of the medical profession and the core obligation of medical practice and also safeguards her integrity as a medical professional.

Physician A’s objection is not an instance of CO. Her refusal is a profession-based refusal, a refusal grounded in professional norms and standards, which is distinguishable from a conscience-based refusal. Her refusal of the patient’s request appeals, not to conscience, to her core moral beliefs external to the medical profession, but to the moral norms internal to the medical profession. She reasons that providing the service would be incompatible with the core obligation of medical practice, which she grounds in the primary objective of the medical profession, and thus would violate her integrity as a medical professional. So, her refusal is not conscience-based but profession-based and is, evidently, compatible with fulfilling professional obligations.

### A case of conscience-based refusal

Consider Physician B. As a moral agent, he has a core set of moral beliefs that includes ‘It is morally wrong to knowingly and willfully harm others’ and, on the ground of that core belief, is committed to ‘Do not harm other persons’ as a fundamental moral duty. As a medical professional, he grounds the core obligation of medical practice—'Do not harm patient health’—in that fundamental duty, which is grounded in his core belief, as applied to medical practice.

Physician B receives a patient’s request for a legal and professionally accepted service within the scope of his professional competence, such as one of the patient requests in the examples above. Considering the impermissibility principle, he judges that providing the requested service would fail to satisfy the necessary conditions for the permissible violation of the core obligation of medical practice—and, thus, judges that providing the requested service would be incompatible with his core moral belief. Invoking the objectionability principle, he concludes that objection to the patient’s request is morally permissible and, grounding his objection in his fundamental duty, refuses to provide the requested service. He claims that his refusal upholds his core belief and fundamental duty and thus safeguards his integrity as a moral agent.

Physician B’s refusal is an instance of conscience-based refusal, not a profession-based refusal. He has a set of core moral beliefs; he judges that the requested service is incompatible with his core moral beliefs; and he grounds his refusal in his core moral beliefs, claiming that it is conscience-based. His refusal fits the definition, and thus is an instance, of CO.

### Compatibility of conscientious objection with professional obligations

The crucial question regarding whether IT is true, then, is whether Physician B’s CO is compatible with fulfilling professional obligations. I argue that it is.

Physician B’s conscience-based refusal and Physician A’s profession-based refusal share several features directly relevant to the question of compatibility with fulfilling professional obligations: first, both physicians consider the patient’s request according to the impermissibility principle; second, both physicians judge that the requested service is morally impermissible by the same criteria—because implementing the medical intervention would fail to satisfy the necessary conditions for the permissible violation of the core obligation of medical practice; and, third, both physicians invoke the objectionability principle in coming to the same conclusion that refusal is morally permissible. Because of these features, moreover, Physician A’s profession-based refusal is, evidently, compatible with fulfilling professional obligations. It is reasonable to infer, therefore, that Physician B’s conscience-based refusal, like Physician A’s profession-based refusal, is also compatible with fulfilling professional obligations. I thus argue by analogy:

- *Premise 1*: Physician A’s profession-based refusal and Physician B’s conscience-based refusal share several features (enumerated above) directly relevant to fulfilling professional obligations.
- *Premise 2*: Physician A’s profession-based refusal, because of those features, has the additional feature of compatibility with fulfilling professional obligations.
- *Conclusion*: Likewise, therefore, Physician A’s profession-based refusal also has the feature of compatibility with fulfilling professional obligations.

I have thus demonstrated an instance of CO in medical practice that is compatible with fulfilling professional obligations. IT claims that no instance of CO in medical practice is compatible with fulfilling professional obligations. Therefore, IT is false.

### Implications and qualifications

The foregoing argument demonstrates that conscience-based refusals grounded in a core belief of personal morality (‘Do not harm other persons’) can be analogous to profession-based refusals grounded in a core obligation of medical practice (‘Do not harm patient health’) and thus can be compatible with fulfilling professional obligations in medical practice. My argument also demonstrates that CO in medical practice can be reasonably defended without appealing to religious claims as the source of moral beliefs, or to metaphysical claims about the nature and sanctity of conscience, or to political theories about individual rights and liberties.

Moreover, because the foregoing argument demonstrates that IT is false, it logically entails that both the impermissibility syllogism and the unacceptability syllogism—each premised on IT—are unsound. My argument thus refutes the claims made by some prominent physicians and bioethicists not only that conscientious objection is fundamentally incompatible with fulfilling professional obligations but also that conscientious objectors are generally unacceptable within the medical profession.

However, while demonstrating an instance of CO that is compatible with fulfilling professional obligations suffices to refute IT and other claims premised on IT, my defense of CO is modest in its implications and does not address important questions. First, from the fact that conscience-based refusal justified by this one core moral belief is compatible with fulfilling professional obligations, it does not follow that conscience-based refusals justified by any and every core moral belief would be compatible with fulfilling professional obligations. Thus, my argument here would not excuse refusals justified by core beliefs based in invidious discrimination or irrational assumptions.

Second, simply demonstrating that CO can be compatible with fulfilling professional obligations leaves open what conscientiously objecting medical practitioners owe to their patients, colleagues, and employers. Thus, it might be argued, consistently with the argument I have presented here, that, at least in some circumstances, a medical practitioner refusing to provide a service based on conscience still has a duty to inform their patients of available medical interventions and refer their patients to other medical providers. My defense of CO in the medical profession is thus consistent with the compromise position in the CO debate.

## Objections and responses

The conscience incompatibilist wanting to defend IT against refutation might make three objections to the foregoing argument. Insofar as the argument claims to demonstrate an instance of CO that is compatible with fulfilling professional obligations, one might challenge this claim at either end: either this instance of CO is not compatible with fulfilling professional obligations, or this refusal is not an instance of CO. One might also challenge the principles assumed as premises of the argument.

### First objection: physician B’s CO is incompatible with fulfilling professional obligations

First, the conscience incompatibilist might object that this instance of CO is not, in fact, compatible with fulfilling professional obligations. There are two versions of this objection: one might argue that Physician B’s conscience-based refusal is disanalogous to Physician A’s profession-based refusal; or one might argue that refusals compatible with fulfilling professional obligations must be ultimately justified by norms and standards internal to the medical profession.

### Physician B’s refusal is disanalogous to physician A’s refusal

The first version of this objection addresses the analogy and appeals to a disanalogy between the cases to undermine the conclusion. One might observe that there is a relevant difference between Physician A’s refusal and Physician B’s refusal, namely in their respective groundings of the impermissibility and objectionability principles on which each bases their respective refusals. Physician A grounds those principles in the core obligation of medical practice, while Physician B grounds those principles in his fundamental obligation as applied to medical practice. This difference is made manifest in their respective claims by which they ultimately justify their refusals: whereas Physician A ultimately justifies her refusal by the claim of incompatibility with the primary objective of medical practice and her integrity as a medical practitioner, Physician B ultimately justifies his refusal by the claim of incompatibility with his core moral belief and his integrity as a moral agent. This difference, the objection argues, counts against the analogy and weakens the conclusion that Physician B’s conscience-based refusal is compatible with fulfilling professional obligations.

This difference, however, is just the distinction between a profession-based refusal and a conscience-based refusal. Counting that difference against the analogy would effectively assume that the mere fact that a refusal is conscience-based counts against its compatibility with fulfilling professional obligations. That assumption, obviously, would beg the question in favor of the conscience incompatibilism position.

### Physician B’s refusal fails to fulfill professional obligations

The second version of this objection sets aside the analogy and argues from the nature of professional obligation. One might argue that if a refusal is justified by beliefs external to the medical profession, then for that very reason the refusal is incompatible with fulfilling professional obligations. A refusal compatible with fulfilling professional obligations, one might argue, must be ultimately justified by norms and standards internal to the medical profession.

This objection claims, in effect, that only profession-based refusals can be compatible with fulfilling professional obligations. To warrant that claim one would need to make a further meta-ethical claim that medical practitioners have a professional obligation to ultimately justify the moral principles of medical practice only by norms and standards internal to the medical profession. According to that meta-ethical claim, Physician B, because he justifies the moral principles on which he bases his conscientious refusal ultimately on beliefs external to the medical profession, fails to fulfill his professional obligations. But that meta-ethical claim effectively excludes conscience-based refusals, as defined by Wicclair, from compatibility with fulfilling professional obligations from the start. And it implicitly invalidates externalist approaches to justifying the principles of medical ethics, such as the approach of Beauchamp and Childress [7]. Thus, without a robust argument supported by independent premises to warrant the meta-ethical claim, this version of the objection begs the question in favor of the conscience incompatibilism position.

This objection, in both variations, also leads to what seems to me an absurd outcome. It implies that, while Physician A fulfills her professional obligations by refusing a patient’s request because acting on the request would require impermissibly violating a core obligation of medical practice, Physician B fails to fulfill his professional obligations by refusing a patient’s request because acting on the request would require impermissibly violating a core obligation of medical practice. According to this objection, the same refusal for the same reason would both fulfill and fail to fulfill a medical practitioner’s professional obligations.

### Second objection: physician B’s refusal is not an instance of CO

Second, the conscience incompatibilist might object that this instance of refusal is, in fact, not an instance of CO. There are three versions of this objection: one might argue either that Physician B’s refusal is, in fact, an instance of profession-based refusal; or one might argue that Physician B’s refusal is an insignificant instance of conscientious objection; or one might argue that Physician B’s refusal is not, in fact, justified by a core moral belief.

### Physician B’s refusal is not a conscience-based refusal

The first version of this objection observes that Physician B could, hypothetically, have made a profession-based refusal: like Physician A, he could have based his objection on the core obligation of medical practice and justified his refusal by the primary objective of the medical profession. One might thus argue that if a refusal could be profession-based, justified by norms and standards internal to the medical profession, then the refusal is not conscience-based, despite what the refusing medical practitioner claims. Thus, Physician B’s refusal is, in fact, a profession-based refusal, despite his external justification of refusal by his core moral beliefs.

Rather than appealing to a disanalogy between the physicians’ respective refusals, this objection appeals to the analogy itself as an argument against the compatibility of conscience-based refusal with fulfilling professional obligations. The objection claims, in effect, that a refusal is not conscience-based if it is analogous to a profession-based refusal. Thus, Physician B’s refusal is not, in fact, a conscience-based refusal precisely because it is analogous to Physician A’s profession-based refusal.

This objection implicitly assumes that conscience claims, to be conscience-based, must conflict with profession-based claims. That assumption entails altering Wicclair’s definition of a conscience-based refusal to include a fourth provision: (4) the agent’s refusal is at variance with judgments based on professional norms and standards. On this altered definition, Physician B’s refusal is conscience-based only if it disagrees with Physician A’s professional-based refusal; because Physician B’s refusal agrees with Physician A’s refusal, his refusal is not conscience-based.

Altering the definition of a conscience-based refusal to include the necessity of variance with judgments based on professional norms and standards, however, effectively rules out conceptions of medical professional judgment as inherently involving exercise of conscience, such as the view advocated by Symons [2]. The altered definition would not be neutral between the different positions on the question but would be biased in favor of the incompatibilism position. This version of the objection thus begs the question.

### Physician B’s refusal is not a significant instance of conscience-based refusal

The second version of this objection argues that this refusal, while an instance of conscience-based refusal, is not a significant instance of CO because it occurs in non-controversial circumstances. In each example described in the section "Argument: conscientious objection is compatible with professional obligation", the requested medical intervention, although clearly harm-causing/risking, was not clearly medically indicated for the patient’s condition. The CO debate is about whether a medical professional may refuse a patient’s request for a medical intervention that is legal, professionally accepted, within the scope of their professional competence, *and medically indicated*. Because the latter condition is not the case in the examples described, the argument by analogy proves at most a trivial result: every physician would (or could) reasonably object to the patient’s request, so nothing significant for the CO debate is really proved here.^11^

This version of the objection implies altering Wicclair’s definition of conscientious objection to include a further condition: “A medical professional engages in an act of conscientious objection when they: (1) refuse to provide legal and professionally accepted goods or services within the scope of their professional competence *that are medically indicated for the patient’s condition*…” The question is whether this is a reasonable alteration to Wicclair’s definition. This alteration implies that refusal of a medically indicated intervention is a necessary condition of a significant instance of CO: if a patient-requested intervention is not medically indicated, then a physician’s refusal of that intervention cannot be a significant instance of CO. I think there is reason to question this alteration, which can be demonstrated by counterinstance.

Consider patient requests for abortion in situations parallel to the first and second examples described in the section "Preliminaries: aims, means, definitions, and principles". A pregnant patient requests an abortion because, say, they believe they will have a happier life without a child or because they fear for the future of the planet due to overpopulation or because they do not want to expose a child to the cruel evils of this world. Or a pregnant patient requests an abortion because, say, their previous pregnancy developed serious complications dangerous for the fetus and their health and they worry that this pregnancy will likewise develop serious complications dangerous to either the fetus or their health, even though the patient appears in complete health and there is no empirical evidence of any medical complication with this pregnancy. Now, abortion is a legal and professionally accepted medical intervention that also causes/risks harm to the patient’s health.^12^ According to the Permissibility Conditions and Objectionability Principle, a physician may refuse both patient requests for the reasons of lack of a health aim (Condition 1) in the former situation and lack of a demonstrated health need (Condition 2a) in the latter situation. As argued in the second section above, these refusals could be grounded in the core belief and fundamental obligation of personal morality, such that these refusals could count as conscience-based refusals.

The question relevant to the present objection is whether these conscience-based refusals count as significant instances of CO. According to the altered definition of CO implied by the objection, they do not count as significant because abortion is not medically indicated in either situation. Yet, it seems to me, at least some parties to the debate over CO and abortion would consider these as significant instances of CO. In fact, prominent conscience incompatibilists have argued that because the medical profession considers abortion to be a standard intervention, any physician’s conscience-based refusal to fulfill a patient’s reasoned request for a legal abortion, even for a patient reason the physician considers a lifestyle choice, is unjustifiable and impermissible [5, 10]. Such views imply that any physician conscience-based refusal of a patient’s reasoned request for a legal abortion, even if not medically indicated, is significant for the CO debate. Thus, not every instance of significant CO requires refusal of a medically-indicated intervention.

Nonetheless, for the sake of discussing the objection, if one accepts this alteration to Wicclair’s definition, then the question is whether one could demonstrate an instance of CO based on the core belief (‘It is morally wrong to knowingly and willingly harm another person’) under this further condition that is compatible with fulfilling professional obligations. I think such an instance of CO could be demonstrated.

To demonstrate such an instance of CO, I will augment the fundamental obligation of personal morality and core obligation of medical ethics as set forth in the second section above by adding a further reasonable condition to each principle. I will augment the criteria for permissibly violating the fundamental obligation of personal morality (“Do not harm others”) by adding a further criterion: An action to help another person in need that foreseeably would result in harm to others is morally permissible only if…*the benefit outweighs the harm*. Similarly, I will augment the core obligation by adding to the Permissibility Conditions a fourth necessary condition:

2. Some harm resulting from medical intervention to benefit patient health is practically unavoidable as judged by medical expertise, including at least the following…

*(d) Proportionality of health benefit: the expected benefit to patient health outweighs the harm caused/risked to patient health*.

Like the other three necessary conditions, this fourth necessary condition could be grounded either externally in the core belief and fundamental duty of personal morality, or internally in the primary objective and core obligation of the medical profession.

Now I will describe a case that fits the added condition, referring to Physician A and Physician B from the previous section. Patient E visits Physician B complaining of severe abdominal pain. Examining the patient, Physician B discovers a large bulge in the abdominal wall and diagnoses a ventral hernia. Considering the severity of the hernia, Physician B judges that the risk of intestinal damage and other serious complications from the hernia indicate that surgery is needed to repair the hernia. The patient agrees with Physician B’s recommendation and requests surgery. Upon checking the patient’s medical chart, however, Physician B learns that the patient has several serious comorbidities and thus determines that the patient would face a significant risk of not recovering from the surgery or even not surviving the surgery. Physician B judges that, although the patient’s request is health-aimed and the patient has a diagnosed hernia, and although surgery would be reasonably expected to alleviate the hernia and the hernia cannot be alleviated without surgery, the expected health benefit of surgery would not outweigh the expected health harm caused/risked by surgery. Physician B thus judges that, considering the patient’s overall health condition, while the requested harm-causing/risking medical intervention meets necessary conditions (a)–(c), it fails to meet necessary condition (d), such that performing the hernia surgery is impermissible and objection is permissible.

Physician B explains the risks to Patient E and advises against the surgery, at least until the patient’s comorbidities are resolved, recommending non-invasive measures to prevent the hernia from worsening and reduce discomfort in the meantime. The patient, however, disagrees with Physician B’s revised recommendation: rationally considering their personal preferences, the patient states that they would rather take the risk of not recovering or not surviving to avoid having to bear the burden of the hernia. The patient thus persists in requesting immediate surgery. Physician B refuses the patient’s request, stating that he could not proceed with the surgery in good conscience because it would require him to impermissibly violate the “Do not harm” principle and thus act against his core beliefs. But Physician B offers to refer the patient to another physician for a second opinion.^13^ Patient E visits Physician A on referral from Physician B. Physician A consults Physician B’s notes, examines the patient, and reviews the patient’s medical chart. Physician A judges that, despite the patient’s comorbidities, the health benefits of surgery would outweigh the health risks, such that condition (d) is met. Physician A thus agrees to the patient’s request for immediate surgery.

Here I have demonstrated a case involving a conscience-based refusal of a harm-causing/risking intervention that is medically indicated for the patient’s condition. According to the altered definition of CO, this case counts as a significant instance of CO. The question then is whether Physician B’s conscience-based refusal of Patient E’s request is compatible with fulfilling professional obligations. I argue that it is. Physician B exercised sound medical judgment in coming to his conclusion on the permissibility of surgery: he carefully considered all the relevant medical facts of the patient’s condition; he coherently applied the relevant medical ethical principle to the medical facts; he explained to the patient his reasons for recommending against surgery, based on medical facts and ethical principle; and he recommended to the patient a treatment option appropriate to the patient’s medical condition. Moreover, Physician B acted on the core obligation of medical practice in pursuit of the primary aim of the medical profession, maintaining and restoring the patient’s health. Physician B, therefore, fulfilled his professional obligation to Patient E.

Now, the objector might reply that Physician A could have come to the same conclusion as Physician B, that the health benefits of surgery would not outweigh the health harms caused/risked by surgery, and thus recommended against surgery and then refused Patient E’s request for surgery. The only difference between Physician A’s agreeing to Patient E’s request and Physician B’s refusing of Patient E’s request was a difference of professional judgment regarding the weighing of benefits and harms. Because Physician A could have similarly made a reasonable refusal of Patient E’s request had she judged the medical facts differently, this case also is insignificant for the CO debate.

At this point, this version of the objection collapses into the previous version of the objection, requiring that a conscience-based refusal must be at variance with judgments based on professional norms and standards. And I have already argued that the previous version of the objection begs the question.

### Physician B’s refusal is not justified by a core belief

The third version of this objection argues that this refusal is not justified by a core moral belief because the moral belief in view—'It is morally wrong to knowingly and willfully harm another person’—is not, in fact, a core belief. One might argue this point in either of two ways.

One might argue that this moral belief is not a core belief of any one person because it is a common belief of most reasonable people. Core beliefs are the subset of beliefs mattering most to a person, and what matters most to a person is not something shared with most other people.

This way of putting this objection implicitly assumes that core beliefs must be idiosyncratic to the person. That assumption entails altering Wicclair’s definition of a core belief to include the feature of being idiosyncratic. But that alteration of definition seems to require too much for a belief to count as a core belief and, in effect, excludes beliefs that surely seem to count as core beliefs. Consider this belief: ‘Every human life is of equal value and deserves respect because all human beings are created in the image of God.’ That belief is shared by two billion Christians, and most of them I expect would say that they consider this to be one of their core moral beliefs. Consider also the patriotic beliefs of typical citizens of countries around the world. Many people would consider the belief that one has a duty of loyalty to one’s country to be a core moral belief, despite sharing that belief with millions of people.

One might argue instead that, while common beliefs can be core beliefs, *this* moral belief is not a core belief. This belief does not belong to the subset of beliefs that matter most to a person, that are integral to a person’s identity and integrity, such that acting contrary to this belief is perceived by the person as an act of self-betrayal.

I think this moral belief is a core belief and that there is empirical evidence to support this claim. Consider Stanley Milgram’s infamous obedience-to-authority experiments. The participants *believed* that they had knowingly and willfully harmed the other person in the experimental set-up without actually having caused any harm. Many participants were visibly distressed during the experiment, and some participants suffered lasting distress and even trauma, despite afterward receiving reassurances from the experimenters that the shocks were not as painful as it seemed and that sometimes some people need to be harmed for their own good [11]. This psychological harm suggests that these participants perceived their apparently other-harming actions as integrity-violating and self-betraying.

Consider training soldiers to kill in battle. Research by Lt. Col. David Grossman (U.S. Army, Ret.) details how U.S. combat training changed during World War II to accommodate the demonstrable fact that human beings have a natural resistance to killing fellow humans. Combat training would thus have to overcome this resistance: the soldier must be habituated to killing, and the enemy must be dehumanized [12]. The psychological reality that becoming efficient at killing humans requires re-habituating the self also suggests that people perceive actions to knowingly and willfully harm others as identity-altering and self-betraying.

### Third objection: the argument begs the question and so fails to demonstrate its conclusion

Third, the conscience incompatibilist might argue that my argument begs the question by the principles on which it is premised. Those principles make nontrivial claims about the normative nature of the medical profession, the moral priority of medical practice, and the nature of health and harm. They assume that the medical profession aims primarily at patient health only, that professional obligation prioritizes avoiding harm to patient health over satisfying patient preferences, and that refusing a patient’s request does not by itself harm the patient’s health (even if the patient’s desires are frustrated).

Were it assumed instead that the primary objective of the medical profession is the more comprehensive goal of patient *wellbeing*, that the moral priority of medical practice is respecting patient *autonomy*, and that refusing the patient’s reasoned request could constitute *harming* the patient even if the patient’s health is not harmed, then it could be argued that violating the professional obligation ‘Do not harm patient health’ would be permissible on the ground of the patient’s informed and reasoned consent to the harm-causing/risking medical intervention. Under these alternative principles, the physician’s duty to satisfy the patient’s autonomous decisions would be a strong moral consideration sufficient to override the physician’s duty to not harm patient health. If so, then there would not be a profession-based ground to justify refusal in the cases constructed, such that it would not follow that a conscience-based refusal is analogous with a profession-based refusal and thus is compatible with fulfilling professional obligations. The argument, therefore, fails to demonstrate that IT is false.

This seems to me the objection most deserving of serious consideration. This objection carries two important implications for the CO debate in the medical profession. First, it exhibits the fact that *the incompatibilism position depends on normative assumptions about the medical profession, physician obligation, and health and harm*. These are nontrivial assumptions that are often disputed by conscience compatibilists and thus must be supported with substantive arguments by conscience incompatibilists. Second, it demonstrates that the CO debate in the medical profession is not about whether exercising conscience is compatible with fulfilling professional obligations, but rather about the normative assumptions—concerning the nature and aim of the medical profession, the role and responsibility of medical professionals, and the moral and empirical aspects of medical judgment—that should govern the medical profession.

## Footnotes

1 The CO defender could dispute premise 1, arguing that voluntarily joining the medical profession does not necessarily entail such an obligation on the medical professional. But, because premise 1 is endorsed by major supporters of IT, it strengthens the refutation of IT to allow this premise here: my refutation of IT is logically independent of whether premise 1 is true. For challenges to premise 1, see [1–3].

2 The third sentence in the long quotation (“If people…”) is effectively equivalent to premise 1 of the unacceptability syllogism.

3 For example, a physician, acting by consent of a patient’s designated health care proxy, might administer a treatment that improves the patient’s condition but which the patient had previously expressly refused.

4 This is a minimal set of criteria. Further criteria might be added, such as the harm is only a result of the action and not the means to the benefit and the benefit outweighs the harm, the latter of which I will consider in the final section. I formulate these criteria as necessary conditions, not as a set of necessary and sufficient conditions. I thus leave it undetermined whether meeting both criteria (or both criteria plus further criteria) renders the action permissible. It suffices for the argument presented that failure to meet either criterion renders the action impermissible. The argument presented here, therefore, does not depend on commitment to either a particular version of the principle of double effect (a complete set of necessary and sufficient conditions) or a fully-developed theory of moderate deontology.

5 Further core obligations derived from the primary objective could be enumerated, of course, such as ‘Act to benefit patient health.’ The negative duty of not harming patient health, however, is all that is needed for the argument presented. Whether the negative duty of not harming patient health should be considered an always stronger duty than the positive duty of benefiting patient health is an important question of medical ethics but tangential to this paper’s purpose.

6 The externalist approach to justifying a duty of ‘do not harm’ (nonmaleficence) in medical practice is followed, for example, by Beauchamp and Childress in [7].

7 This is a minimal set of criteria. Further criteria could be added, such as the patient’s (or their proxy’s) informed consent and the health benefit expected outweighs the health harm risked, the latter of which we’ll consider in the final section. Again, I formulate these criteria as necessary conditions, not as a set of necessary and sufficient conditions. I thus leave it undetermined whether meeting both criteria (or both criteria plus further criteria) renders the medical intervention morally permissible. It suffices for the argument presented that failure to meet either criterion renders the medical intervention morally impermissible.

8 One could reasonably argue for the stronger principle that it is not merely permissible but obligatory for a medical professional to object/refuse when a patient’s request for a harm-causing/risking intervention fails to meet these necessary conditions. However, a weaker principle of permissibility suffices for the argument presented.

9 The health harms and risks associated with appendectomy include incision, bleeding, wound infection, blocked bowel, and injury to nearby organs [8].

10 Whether the physician has a duty of referral when refusing the patient’s request in any of these examples is an important question in the CO debate but tangential to this paper’s purpose.

11 This objection was raised by an anonymous reviewer.

12 The situations described assume that the patient lives and the physician practices under a broad legal protection for abortion rights. The health risks associated with abortion include heavy bleeding, blood clots, womb infection, sepsis, uterine perforation, cervical injury, and incomplete abortion [9].

13 Again, whether Physician B has a duty to refer Patient E is an important question in the CO debate but tangential to responding to the objection.
